# Link-based quantitative methods to identify differentially coexpressed genes and gene Pairs

**DOI:** 10.1186/1471-2105-12-315

**Published:** 2011-08-02

**Authors:** Hui Yu, Bao-Hong Liu, Zhi-Qiang Ye, Chun Li, Yi-Xue Li, Yuan-Yuan Li

**Affiliations:** 1 Bioinformatics Center, Key Laboratory of Systems Biology, Shanghai Institutes for Biological Sciences, Chinese Academy of Sciences, 320 Yueyang Road, Shanghai 200031, P. R. China; 2 Graduate University of the Chinese Academy of Sciences, 19A Yuquanlu, Beijing 100049, P. R. China; 3 Shanghai Center for Bioinformation Technology, 100 Qinzhou Road, Shanghai 200235, P. R. China; 4 School of Life Science and Technology, Tongji University, 1239 Siping Road, Shanghai 200092, P.R. China; 5 Department of Biostatistics, Vanderbilt University School of Medicine, Nashville, TN 37232, USA; 6 Center for Human Genetics Research, Vanderbilt University School of Medicine, Nashville, TN 37232, USA

## Abstract

**Background:**

Differential coexpression analysis (DCEA) is increasingly used for investigating the global transcriptional mechanisms underlying phenotypic changes. Current DCEA methods mostly adopt a gene connectivity-based strategy to estimate differential coexpression, which is characterized by comparing the numbers of gene neighbors in different coexpression networks. Although it simplifies the calculation, this strategy mixes up the identities of different coexpression neighbors of a gene, and fails to differentiate significant differential coexpression changes from those trivial ones. Especially, the correlation-reversal is easily missed although it probably indicates remarkable biological significance.

**Results:**

We developed two link-based quantitative methods, DCp and DCe, to identify differentially coexpressed genes and gene pairs (links). Bearing the uniqueness of exploiting the quantitative coexpression change of each gene pair in the coexpression networks, both methods proved to be superior to currently popular methods in simulation studies. Re-mining of a publicly available type 2 diabetes (T2D) expression dataset from the perspective of differential coexpression analysis led to additional discoveries than those from differential expression analysis.

**Conclusions:**

This work pointed out the critical weakness of current popular DCEA methods, and proposed two link-based DCEA algorithms that will make contribution to the development of DCEA and help extend it to a broader spectrum.

## Background

Identification of differentially expressed genes (DEGs) is a key step in comprehending the molecular basis of specific biological processes and screening for disease markers. This methodology looks at absolute changes in gene expression levels, and treats each gene individually. However, genes and their protein products do not perform their functions in isolation, but in coordination [1], and the dynamic switch of a gene from one community to another always implies altered gene function [2,3]. Therefore, gene coexpression analysis was developed to explore gene interconnection at the expression level from a systems perspective [4-10], and 'differential coexpression analysis (DCEA)', as a complementary technique to the traditional 'differential expression analysis' (DEA) [11,12], was designed to investigate molecular mechanisms of phenotypic changes through identifying subtle changes in gene expression coordination [11-14].

**Figure 1:**
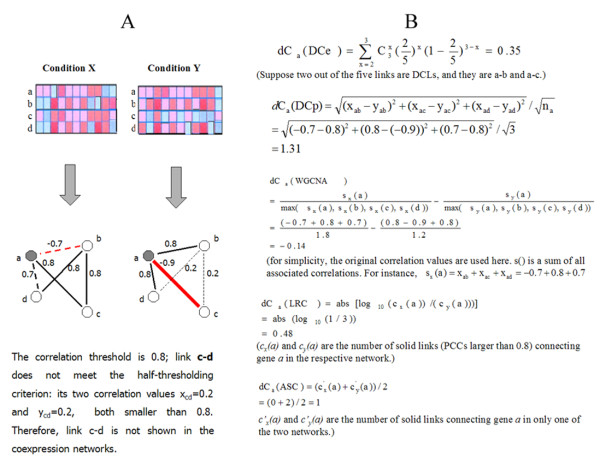
**A simplified illustration of the framework of five DCEA methods**. A), two expression data matrices from two contrastive experimental conditions (X and Y) involving genes a, b, c, and d, visualized using shades from red (high expression level) to blue (low expression level), are transformed to a pair of coexpression networks. In the coexpression networks, gene pairs with absolute expression correlation values larger than 0.8 are connected with solid lines, while the rest with dashed lines. The line thickness is proportional to the absolute coexpression value. Red color highlights a negative coexpression value, and the grey-shaded node, gene a, is the one whose differential coexpression (*dC*) calculations are to be illustrated. B), different DCEA methods calculate the *dC *measure of gene a in different ways (see Results and Discussion for details).

In a typical DCEA workflow, a pair of gene expression datasets under two conditions, such as disease and normal, are transformed to a pair of coexpression networks in which links represent transcriptionally correlated gene pairs (Figure 1A), and then the differential coexpression is calculated for each gene (Figure 1B). After surveying three previously proposed DCEA methods (Figure 1B): 'Log Ratio of Connections' (LRC) [15], 'Average Specific Connection' (ASC) [12], and 'weighted gene coexpression network analysis' (WGCNA) [16-19], we realize that although DCEA methods have been used more and more frequently in transcriptome studies [11,12,15,17,20,21], they have not been well developed, and the most crucial issue in DCEA - the choice of differential coexpression measure, is far from settled.

In LRC [15], the differential coexpression of a gene is defined as the absolute logarithm of the ratio of its two connectivities - the numbers of links connecting the gene in two coexpression networks. This method does not distinguish the coexpression neighbors of a gene, and hence may fail if the connectivities of a gene in two networks are close while the gene neighbors are rather different. This defect is overcame in the average specific connection (ASC) method [12], which compares the 'specific connections' that exist in only one network. In simply dealing with the numbers of neighboring genes, however, both LRC and ASC fail to achieve a more precise characterization of differential coexpression that would be attainable if the quantitative expression correlation values were not discarded. The third method, WGCNA [16-19], goes beyond ASC and LRC as it compares the sums of expression correlation values associated with a gene between two conditions, which is essentially the comparison of weighted connectivities of a gene. We therefore classify all these three methods into a gene connectivity-based type. Because these connectivity-based methods do not quantify coexpression changes link by link, they cannot precisely estimate the differential coexpression of a gene. As a result, they fail to distinguish dramatically changed links from those relatively trivial ones, and they also cannot detect a special type of coexpression change - correlation reversal between positive and negative, which is never rare [22,23] and probably has important biological significance [24,25].

Since coexpression is in essence a property of gene pairs (links), it should be more reasonable to design link-based DCEA methods that concentrate directly on the coexpresssion change of each gene pair. In this work, we develop two link-based DCEA algorithms for identifying differentially coexpressed genes (DCGs) and differentially coexpressed gene pairs or links (DCLs). Based on the exact coexpression changes of gene pairs, these methods take into account both the gene neighbor identity information and the quantitative coexpression change information. It was demonstrated on simulated datasets that both novel methods had an improved performance over the existing methods to retrieve predefined differentially regulated genes and gene pairs. We furthermore applied the methods to a publicly available expression dataset on type 2 diabetes (T2D) and provided additional information to characterize T2D-related genes. The novel methods for DCEA analysis have been implemented in an academically available R package DCGL [26].

## Results and Discussion

### Novel 'half-thresholding' strategy in constructing gene coexpression networks

There are currently two accepted strategies, namely hard-thresholding [11,12] and soft-thresholding [16-19], for inferring gene coexpression network from expression correlation values. The hard-thresholding one, adopted by LRC and ASC, keeps a link in the coexpression network as long as the coexpression value exceeds a predefined threshold (solid lines in Figure 1A). The soft-thresholding strategy, adopted by WGCNA, keeps all possible links and raises the original coexpression values to a power 'beta' so that the high correlations are emphasized at the expense of low correlations (its formula in Figure 1B uses the untransformed correlation values for illustration convenience). Note that the coexpression value pair associated with the invisible link c-d in Figure 1A are utilized in the WGCNA *dC *formula (Figure 1B). In effect, while the 'hard-thresholding' strategy dichotomizes the continuous correlation values to be coexpression and non-coexpression, it is robust to minor variations and meanwhile its sensitivity is impaired, as some small coexpression changes (link a-d in Figure 1A, correlation values from 0.7 to 0.8) are treated equally as large ones (link b-d in Figure 1A, correlation values from 0.8 to 0.2). On the other side, the 'soft-thresholding' strategy can be overly sensitive when using a low soft-threshold (i.e. a low power) since noisy variations are kept in its calculation. One way to get around this is to increase the power. Another way, proposed here, is to devise a novel "half-thresholding" strategy.

**Figure 2:**
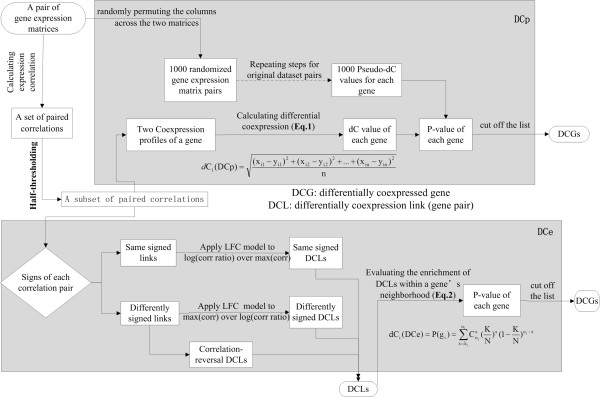
**Flowchart of the main steps involved in the two DCEA methods DCp and DCe**. The upper and lower boxes comprise the major steps for DCp and DCe respectively, while outside the boxes are a few steps of the shared pre-processing process.

With the "half-thresholding" strategy, we keep a link in both coexpression networks if at least one of the two coexpression values exceeds the threshold. In this way, we ignore minor variations of 'non-informative links' whose correlation values in both networks are below the threshold, but thoroughly examine the possibly meaningful coexpression changes of links remaining in the two coexpression networks. Starting with this strategy, we come up with two novel methods for identifying differentially coexpressed genes and/or links from the pair of coexpression networks (Figure 2).

### The 'Differential Coexpression Profile' method (DCp)

We consider two gene expression datasets under two different conditions. For each dataset, we calculate the Pearson correlation coefficients (PCCs) between the expression profiles of all gene pairs. For gene *i *and gene *j*, let *x_ij _*and *y_ij _*denote their PCCs under the two conditions. Then the two datasets are encoded into a set of paired correlations *CP = {(x_ij_,y_ij_)} *over all gene pairs. We then filter out non-informative correlation pairs using the half-thresholding strategy. Specifically, a pair is kept if any of the two PCCs has a q-value lower than a cutoff, say 25%, where the q-value is a false discovery rate estimated from the p-value of PCC using the Benjamini-Hochberg method [27]. This results in a subset of correlation pairs, which are equivalent to two coexpression networks with identical structure but different link weights (PCCs).

For gene *i*, the PCCs between it and its *n *neighbors in the filtered set form two vectors, X = (x_i1_,x_i2_,...,x_in_) and Y = (y_i1_,y_i2_,...,y_in_) for the two conditions, which are referred to as 'coexpression profiles'. We define the differential coexpression (*dC*) of gene *i *with Eq. 1.(1)

This measure captures the average coexpression change between a gene and its neighbors. As this method is based on the differential coexpression profiles, it is denoted as DCp. An example calculation of DCp *dC *is shown in Figure 1B.

The *dC *value can be used to rank genes. To evaluate the statistical significance of *dC*, we perform a permutation test, in which we randomly permute the disease and normal conditions of the samples, calculate new PCCs, filter gene pairs based on the new PCCs, and calculate the *dC *statistics. The sample permutation is repeated 1000 times, and a large number of permutation *dC *statistics form an empirical null distribution. The p-value for each gene can then be estimated.

The major steps of the DCp algorithm is outlined in the upper box of Figure 2.

### The 'Differential Coexpression Enrichment' method (DCe)

While DCp takes advantage of the coexpression changes of individual gene links, its final goal is to identify differentially coexpression genes (DCGs). To extend the findings from DCGs to differentially coexpressed links (DCLs), we devise another method, 'Differential Coexpression Enrichment', which first identifies DCLs, and then identifies DCGs. As the method is based on enrichment of DCLs, it is named DCe.

The filtered correlation set (determined with a cutoff ρ of expression correlation values or q_th _of the q values, as described in the DCp method details) represents the beginning links to be screened for DCLs. For a link or a pair of correlation values, we first determine the maximum (absolute) correlation and the log (absolute) correlation ratio. If the two correlation values of a link are same signed, we intuitively propose that the log correlation ratio may serve as a basic measure for the link's differential coexpression; in contrast, if the link has two differently signed correlation values, its differential coexpression is more likely to be reflected by the maximum correlation. We then separately deal with the same signed links and the differently signed links using the limit fold change (LFC) model [28]. LFC is a robust statistical method originally proposed for selecting differentially expressed genes (DEGs), by modeling the relationship between maximum expression and log expression ratio of genes. In coexpression analysis, we instead model the relationship between maximum coexpression and log coexpression ratio of links.

**Figure 3:**
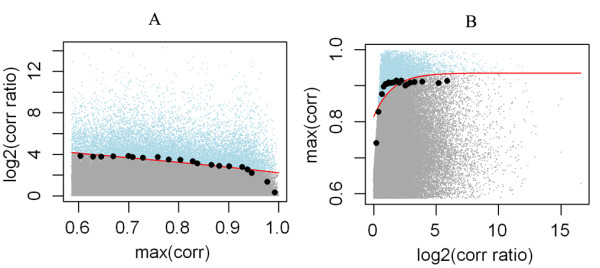
**Limit fold change model applied to identify differentially coexpressed links (DCLs) from a simulated dataset pair in group C (dataset pair III)**. Each point represents a gene pair or a link characterized by its log correlation ratio and maximum absolute correlation value. A curve (red) y = a + (b/x) is used to fit the boundary outliers (black dots) determined by fraction δ, and points (blue) lying above the fitted curves are considered DCLs. A, same signed links; B, differently signed links.

For the same signed links, as is illustrated in Figure 3A, we categorize them into bins according to their maximum coexpression values, and within each bin, select a fraction δ of links with highest log fold changes, and fit a curve y = a + (b/x) over the boundary links. Links lying above the fitted curve are considered as DCLs. In most experiments of this work, we set δ = 0.1, but the effect of tuning δ was tested in the following simulation study.

Among the differently signed links, those with both PCCs surpassing the cutoff ρ of correlation values or q_th _of the q-values are directly taken out as DCLs, specifically, correlation-reversal DCLs. In parallel to the same signed case, LFC model is applied to the remaining differently signed links with the roles of maximum coexpression and log coexpression ratio flipped due to our foresaid consideration (Figure 3B). Again, links above the fitted curve are considered as DCLs.

Suppose there are *N *links in each filtered coexpression network, from which we have determined *K *DCLs using the procedures above. For gene *i *with *n_i _*links of which *k_i _*are DCLs, the p-value is calculated based on a binomial probability model (Eq. 2). The obtained p-value can be regarded as the *dC *measure of a gene, with a smaller value indicating a higher degree of differential coexpression. The enrichment step of DCe method is also illustrated in Figure 1B.(2)

The major steps of the DCe algorithm is outlined in the lower box of Figure 2.

### Comparing different DCEA methods in simulation experiments

In a simulation experiment, we first define two gene regulation networks, which are overall similar but have differences in a small portion of regulation relationships (gene links), then simulate two gene expression datasets based on the two networks, respectively. The predefined discrepant regulations are termed differentially regulated links (DRLs) and the associated genes are differentially regulated genes (DRGs). We evaluated DCp and DCe in terms of their capability to retrieve the predetermined DRGs and DRLs from the simulated data. Also included in the comparative evaluation were three representative DCEA methods that we reviewed in the Background: LRC [15], ASC [12], and WGCNA [16,19]. Note that the WGCNA has evolved into two slightly different versions, the 'signed' and the 'unsigned', and here we adopted the signed version and set its parameter beta at the default 12.

We first analyzed a pair of simulated datasets (dataset pair Z) from a published study [29], which were generated based on two yeast signaling networks using SynTReN [30]. A total of seven genes, PHO2, FLO1, MBP1_SWI6, FLO10, TRP4, CLB5 and CLB6, were involved in the altered interconnection [29], therefore taken as DRGs. As Table 1 shows, the DCp *dC *score ranked all seven DRGs exclusively at the top, while the DCe p-value ranked six at the top and the other one at the 8th position; both methods had better performances than the other three methods. It was noticeable that SWI4, a gene falsely detected in the original study [29], puzzled WGCNA and ASC (which both ranked it at the 5^th ^position), but not DCp and DCe (which ranked it at the 9^th ^or 15^th ^position).

**Table 1:** The twenty yeast proteins involved in simulated dataset pair Z and the ranking of them by DCEA methods DCp, DCe, signed WGCNA, ASC, and LRC separately.

	DCEA methods				
	
protein	DCp	Dce	signed-WGCNA	ASC	LRC
**PHO2**	1	1	3	1	5
**MBP1_SWI6**	2	3	8	2	8
**FLO1**	3	2	1	10	7
**FLO10**	4	4	2	6	4
**TRP4**	5	5	4	7	9
**CLB5**	6	6	14	3	18
**CLB6**	7	8	18	4	19
ACE2	8	14	16	15	1
SWI4	9	15	5	5	16
CDC11	10	7	9	12	17
CDC10	11	11	10	13	10
SWI4_SWI6	12	16	6	8	12
HTB1	13	13	7	11	15
ACT1	14	12	13	14	6
CAF4	15	9	19	19	3
LEU2	16	17	11	9	13
SPT16	17	18	15	18	11
HO	18	10	17	16	2
CTS1	19	19	12	17	14
SNF6	20	20	20	20	20

The proteins are sorted by the DCp ranks. Bold refers to the truly differentially regulated genes (DRGs) in the simulation.

Additionally, we used SynTReN to simulate three groups of dataset pairs (denoted data groups A, B, C) based on a predefined E.coli gene regulatory network of a total of 1300 genes [30]. Specifically, we selected a sub-network of 1000 genes as the original network, and exerted artificial perturbation on 10% of its links as if it was from a different condition. The three groups had different perturbation types. For group A, we used regulation-elimination (removing a link between a pair of genes). For group B, we used regulation-switch (switching the regulation effect between activation and repression). For group C, we applied half regulation-elimination and half regulation-switch. For each group, we generated five dataset pairs, one simulated from the original network and the other from the perturbed network.

**Figure 4:**
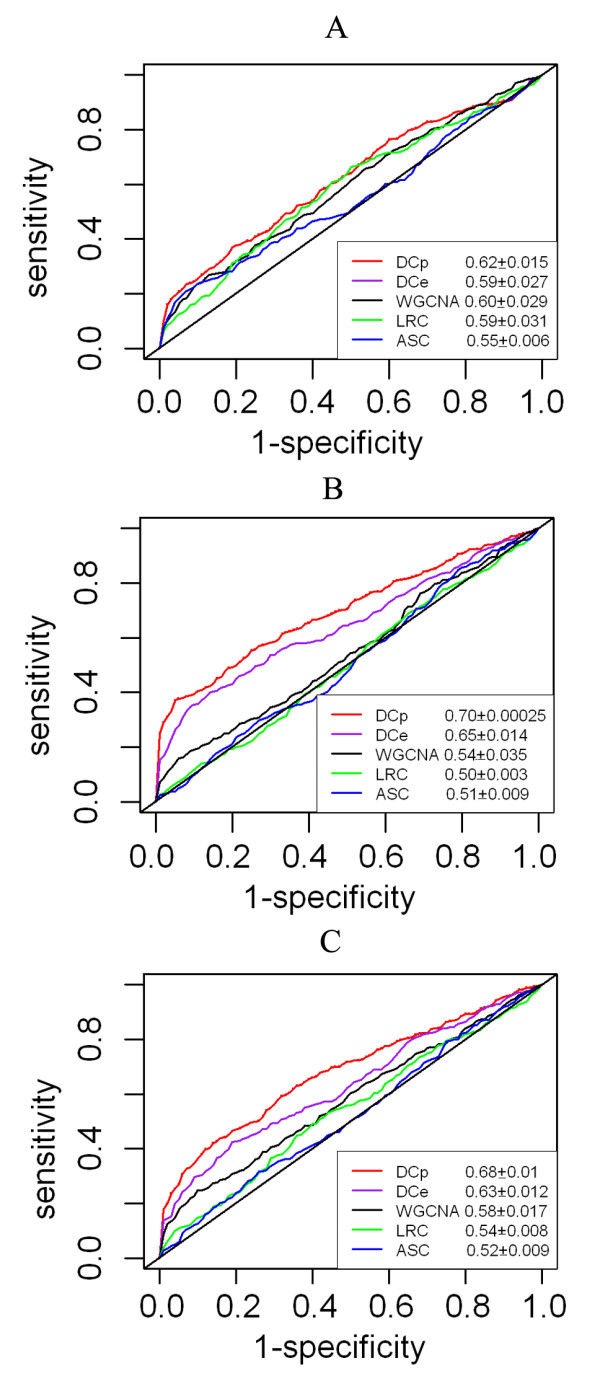
**Receiver-operating-characteristic (ROC) curves showing the capabilities of five DCEA methods in retrieving predefined DRGs**. To simulate change of regulatory relationships, 10% links were removed (A), 10% switched (B), 5% removed and 5% switched (C) in a 1000-node network. Each curve is averaged over five simulations. The numbers in legend are areas under the ROC curves (mean and std).

We applied every DCEA method on every dataset pair and plotted the Receiver-Operating-Characteristic (ROC) curves to show the balance of the five methods between sensitivity and specificity in identifying DRGs (Figure 4). Dataset group A, simulating regulation-elimination, seemed a tough problem for all methods, as none of the ROC curves was obviously far away from the diagonal line representing random assortment (Figure 4A). Nevertheless DCp performed better than the others. The advantage of DCp and DCe over the other methods was increased on group B which simulated regulation-switch, while the performances of ASC and LRC were not significantly different from a random guess (Figure 4B). On dataset group C with both regulation-elimination and regulation-switch included, DCp and DCe still outperformed other methods (Figure 4C). In all, DCp and DCe did better in retrieving DRGs, especially on data involving switched regulation relationships. The WGCNA method, which utilizes the continuous expression correlation values as DCp and DCe do, ranked immediately after DCp and DCe, ahead of LRC and ASC.

Since the signed WGCNA performed better than the other existing methods ASC and LRC and it actually gains more acknowledgement from users, we additionally performed a more comprehensive comparison of WGCNA against the novel methods, with different settings of the key parameter beta of WGCNA taken into account. It turned out that in general the signed WGCNA was more powerful than the unsigned WGCNA, but yet both were incomparable to DCp and DCe regardless of the choices of the parameter beta (Additional File 1: more parameter testing for gene-level evaluation). We also found that the WGCNA methods' performances deteriorate with the beta value, especially when beta exceeded eight, and that the WGCNA methods were relatively more competent for the regulation-elimination scenario (Additional File 1: more parameter testing for gene-level evaluation).

As all these DCEA methods except WGCNA involve a gene link filtering step, or a correlation value thresholding process, we repeated the performance comparison on various q_th _values (0.25, 0.2 and 0.1); additionally, as the perturbation rate of 10% was set arbitrarily, we also tried another two levels, 20% and 30%. It proved that DCp and DCe consistently outperformed the other three with DCp doing better than DCe in most situations (Additional File 1: more parameter testing for gene-level evaluation). We also found that for perturbation rates 10% and 20%, algorithm performances generally increased with more stringent q_th _values, and they dropped a little when the perturbation rate reached 30%. Finally, we tested the sensitivity of algorithms to sample sizes of datasets. At sample size five, six, seven, eight, nine, ten, fifteen and twenty, it was shown that the performances of all algorithms were basically stable, and that DCp and DCe were better than the others (Additional File 1: more parameter testing for gene-level evaluation).

Finally, we compared the only two methods, DCe and ASC, which have the potential to retrieve DCLs, with respect to their capability to retrieve DRLs. It was found that, in three simulated dataset pairs (I, II, and III), DRLs always accounted for a tiny fraction of identified DCLs, but DCe outperformed ASC in enriching DRLs in DCLs (Table 2). As gene coexpression changes may spread from the perturbed links to adjacent ones, we took DRLs and their one-step adjacent links as 'extended DRLs'. Likewise, DCe enriched the extended DRLs (Table 2), which was statistically significant according to tests against empirical distribution of randomly sampled links (Additional File 2: significance in link-level evaluation). In an actual practice of DCe, in order to narrow down the identified DCLs for a follow-up examination, one can raise coexpression value cutoffs (ρ) or lower outlier fractions (δ). We found that raising ρ refined correlation-reversed DCLs efficiently, while lowering δ not only cut down the number of DCLs of the other two types (same sign and different sign) but improved the accuracy of identified DCGs (Additional File 3: reducing DCL scales). Besides, the identified DCLs could also be sifted according to their relevance with a selected gene list, for example, DCGs.

**Table 2:** Fractions of DRLs or 'extended DRLs' in DCL sets.

Golden Standard	Dataset	Background	DCe-DCLs	ASC-DCLs
True	I	8.8e-4	1.5e-3	8.5e-4
DRLs	II	1.0e-3	3.7e-3	7.1e-4
	III	9.8e-4	3.4e-3	7.9e-4

Extended	I	0.44	0.49	0.45
true	II	0.43	0.67	0.43
DRLs	III	0.43	0.63	0.44

I, II, and III denote three dataset pairs from the data groups A, B, and C, respectively.

In summary, the results from simulation studies indicate that reasonably designed DCEA methods can retrieve pre-set differentially regulated genes and links from expression datasets. That is, based on the results from a series of rigorously designed simulation experiments, we provide a preliminary support to the anticipation that DCEA methods are capable of deciphering differential regulation or differential networking underpinning diseases [13].

### Uniqueness of DCp and DCe compared to existing DCEA methods

We attributed the improved performance of DCp and DCe mainly to the exploitation of the linkwise quantitative coexpression changes, which starts with our 'half-thresholding' strategy in coexpression network construction, and continues with the *dC *measures reflecting the linkwise coexpression changes (Eq. 1 and Eq. 2). Capturing the linkwise coexpression changes is much more reasonable than merely extracting the connectivity and/or neighbor identity, or getting the summed correlation values. That is why DCp and DCe outperformed existing methods LRC, ASC, and WGCNA in simulation studies.

We tried designing our methods based on coexpression changes of all possible links, i.e., discarding the half-thresholding, but found the performance was not comparable to the current version of DCp and DCe (data not shown). This suggests the necessity of the link prescreening step. However, it is not easy to determine the optimal coexpression threshold for each specific study, and further investigation on optimizing our half-thresholding procedure is necessary.

It is noticeable that DCp and DCe are especially good at identifying a special type of coexpression change, the coexpression reversal between positive and negative, which is why they have the greatest advantage in the simulated datasets involving regulation-switches. In previous studies, negative correlation values were often flipped to positive values [12], set to zero [31], or crushed to a very narrow region on the right of zero[16,19], and these operations hindered coexpression reversals from emerging. In reality, coexpression reversal probably has biological significance. Taking the coexpression of *p53 *and *Klf4 *as an example, it was recently reported that the positive or negative correlation between these two genes determines the outcome of DNA damage - DNA repair or apoptosis [24]. We believe our attention to this special coexpression change will help to explore subtle mechanisms involved in tuning of molecular balances between opposite factors.

### Re-analyzing a T2D dataset from the perspective of differential coexpression

For an application of the novel DCEA methods, we downloaded dataset GSE3068 from the Gene Expression Omnibus (GEO) database. GSE3068 was designed to study type 2 diabetes (T2D) in rats. It involves 6,955 probesets interrogating 4,765 genes, and the twenty samples herein were divided equally into a T2D group and a normal group. Details on preprocessing this dataset are in "Additional File 4: Preprocessing GSE3068".

We applied DCp to GSE3068 to identify differentially expressed genes (DCGs) and obtained 337 (p-values cutoff 0.05, FDR < 65%) DCGs out of 4765 genes. We listed the 337 DCGs in "Additional File 5: 337 DCGs identified by DCp" regarding their *dC *scores, log fold changes, and potential relevance with T2D (T2D-associated or T2D-related genes are provided in Additional File 6: 52 T2D-associated genes & 425 rat T2D-related genes). The DCGs with T2D relevance deserving more attention were selected and shown in Table 3.

**Table 3:** DCGs with existing evidence of T2D-relevance.

gene	DCP.*dC*	Expression fold change	Reported Relevance
Rapgef4	1.29	0.81	T2D-related
*Nr5a1*	1.28	0.56	T2D-related
*Cd28*	1.21	1.21	KEGG rno04940
*Ucp2*	1.18	1.31	T2D-related
*Pparg*	1.17	1.05	T2D-related; T2D-associated
*RT1-Bb*	1.16	0.74	KEGGrno04940
*Cox6a2*	1.15	0.57	KEGG rno00190
*Bdnf*	1.13	2.13	T2D-related
*Gad1*	1.12	0.23	KEGG rno04940
*Prkaa1*	1.12	1.03	KEGG rno04910
*Prkab1*	1.11	0.52	KEGG rno04910; T2D-related
*RT1-Da*	1.09	1.99	KEGG rno04940
*Nos3*	1.09	0.76	T2D-related
*Cox6c1*	1.09	0.62	KEGG rno00190
*Inpp5d*	1.08	2.36	KEGG rno04910; T2D-related
*Arnt*	1.07	0.71	T2D-related
*Sstr5*	1.06	1.77	T2D-related
*Lipe*	1.06	2.04	KEGG rno04910; T2D-related
*Cacna1a*	1.06	0.55	KEGG rno04930
*RT1-Ba*	1.05	1.71	KEGG rno04940
*Tnfrsf1a*	1.05	1.07	T2D-related
*Il6*	1.05	1.77	T2D-related
*Gip*	1.05	0.63	T2D-related
*Cacna1c*	1.05	0.74	KEGG rno04930
*Mapk10*	1.03	0.79	KEGG rno04930; KEGG rno04910; T2D-related
*Nras*	1.02	0.71	KEGG rno04910
*Serpine1*	1.02	2.07	T2D-related
*Kcnj5*	1.01	0.74	T2D-related
*Hla-dma*	1.01	2.84	KEGG rno04940

Included genes appeared in at least one of the following sources: KEGG T2D-related pathways (Rno04930: type II diabetes mellitus; rno04940: type I diabetes mellitus; rno04910: insulin signaling pathway; rno00190: oxidative phosphorylation); a self-compiled set of 425 T2D-related genes in rat; a list of 52 T2D-associated genes. The lists of "T2D-related" genes and "T2D-associated" genes are provided in "Additional File 6: 52 T2D-associated genes & 425 rat T2D-related genes".

**Figure 5:**
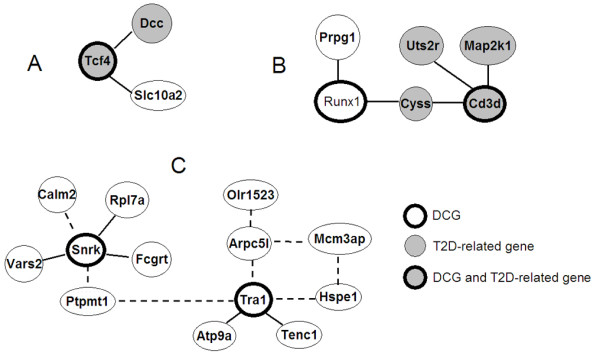
**Genes were organized into network modules via correlation-reversal relationships**. A solid link connects a pair of genes with positive correlation in normal state but negative in disease state, while a dashed link connects genes with negative correlation in normal state but positive in disease state.

We then identified DCLs using DCe methods, and narrowed them down to 4046 DCLs that were connected with the 337 DCGs (Additional File 7: DCLs identified by DCe). As we believed that correlation-reversal was a noteworthy but neglected type of differential correlation, we took a close-up look at those correlation-reversed DCLs. Out of a total of 110 reversed DCLs (Additional File 8: network modules organized by solely correlation reversals), 73 were connected with the 337 DCGs. Figure 5 shows three subnetwork modules organized solely by reversed links. Subnetwork A (Figure 5A) and B (Figure 5B) included quite a number of previously reported T2D-related genes: *Tcf4 *and *Dcc *[32]; *Cd3d *[33], *Uts2r *[34] and *Map2k1 *[35]. Subnetwork C (Figure 5C) was the largest reversed DCL-organized module and it contained an interesting four-gene-circuit (including *Arpc5l, Tra1, Mcm3ap*, and *Hspe1*) of consistent negative-to-positive correlation reversal. Although not being previously reported to be related with T2D, the genes and reversed links included in Figure 5C, as well as other novel cases reported in the supplementary tables of DCGs and DCLs (Additional File 5: "337 DCGs identified by DCp.xls" and Additional File 7: "DCLs identified by DCe.xls"), should receive adequate attention for their distinct traits from the perspective of differential coexpression. Further studies on the transcriptional mechanisms and functional consequences involved in these DCGs and DCLs would be helpful for elucidating how the changed coordination contributes to the pathogenesis of T2D.

Since GSE3068 had been thoroughly analyzed from the differential expression perspective [36], we investigated the relationship between the two gene expression properties, differential expression (DE) and differential coexpression (DC) in this dataset. We first examined the consistency between the DCp-identified 337 DCGs and the 119 previously reported DEGs, of which 36 could be corrected by oral administration of vanadyl sulfate (VS). It was found that the overlapping of DCGs with the 119 DEGs was not significant (hypergeometric test p = 0.22), but that with the 36 VS-corrected DEGs was significant (hypergeometric test p = 0.01). This indicates that differential expression and differential coexpression are somewhat related to each other at least in the T2D context.

We then looked at the disagreement between DCGs and DEGs. A previous differential coexpression analysis on human cancer using the ASC method reported a quite low level of overlapping between DCEA and DEA results (3%) [12]. In our case, DCGs and DEGs had only 3% (DCGs) or 8.4% (DEGs) in common, with the rest majority genes in disagreement. For instance, both *Pparg *and *Tspan8 *had been found to play key roles in T2D pathogenesis [37-39], but they were identified by DCEA and DEA respectively. *Pparg *had an expression fold change of 1.05 in the GSE3068 dataset (Table 3), so its relevance with T2D was not discerned by DEA. From the perspective of DC, however, it stood out since its *dC *value (1.17) was ranked 28th of all 4765 genes. On the contrary, *Tspan8*, with a large expression change (2.3) but a minor coexpression change (*dC *= 0.57), was identified as a DEG but not a DCG.

According to our brief comparison at the gene level, DEA and DCEA are both powerful techniques to find out useful information from expression data. They are significantly related and mutually complementary. Similar conclusion was made at the pathway level based on the observed interplay of gene differential expression and gene differential coexpression in mouse mammary gland tumor [40].

## Conclusions

In this work, we pointed out the critical weakness of current popular differential coexpression analysis methods, and developed two novel link-based algorithms, DCp and DCe. DCp and DCe differ from previous methods primarily in that they are designed to make use of link-specific correlation change values directly while previous methods mainly focuses on the gene connectivity. A novel strategy to filter links in coexpression networks, the half-thresholding strategy, is also proposed as a necessary pre-processing step of the two novel methods.

Based on the results from a series of rigorously designed simulation experiments, we proved that reasonably designed DCEA methods were able to discriminate pre-set differentially regulated genes and links; in another word, we provided a preliminary support to the anticipation that DCEA methods are capable of deciphering differential regulation or differential networking underpinning diseases [13]. Of the five DCEA methods we surveyed, we proved the overall performances of our DCp and DCe against three existing algorithms, and identified WGCNA as the best of the existing three. It is noticeable that while the existing methods were somewhat comparable to link-based methods in case of pure regulation-elimination perturbations, they were significantly outperformed when regulation-switch perturbations were introduced. Regulation-switch is believed to be an relevant phenomenon in fine-tuning of signal transduction [24].

Applying DCp and DCe to a real expression dataset designed for T2D study, we identified 337 DCGs and their associated 4046 DCLs, which may serve as a useful resource for identification and characterization of T2D relevant genes. We also analyzed the relationship between DEA and DCEA in this example, and pointed out that DEA and DCEA are significantly related and mutually complementary techniques to make discoveries from expression data.

Recently, differential coexpression analysis is being appreciated as a significant step towards the differential networking analysis of complex diseases [41], and the area of DCEA is undergoing rapid development as various solutions to set-wise differential coexpression problems are being proposed [20,21,42]. We believe that our methodological improvement will benefit the development of DCEA and help extend it to a broader spectrum of biomedical studies.

## Authors' contributions

HY came up with the main frameworks of the methods, participated in the computational testing and drafted the manuscript. BHL was in charge of the computational coding and testing, and helped drafting the manuscript. ZQY participated in the method design and helped drafting the manuscript. CL supervised the statistical parts of the methods and modified the manuscript. YYL and YXL conceived of the study, and participated in its design and coordination and modified the manuscript. All authors read and approved the final manuscript.

## Links

The Gene Expression Omnibus database http://www.ncbi.nlm.nih.gov/geo/

The DCGL package http://cran.r-project.org/web/packages/DCGL/index.html

## Supplementary Material

Additional file 1 **more parameter testing for gene-level evaluation**. Click here for file

Additional file 2 **significance in Link-level evaluation**. Click here for file

Additional file 3 **reducing DCL scales**. Click here for file

Additional file 4 **Preprocessing GSE3068**. Click here for file

Additional file 5 **337 DCGs identified by DCp**. Click here for file

Additional file 6 **52 T2D-associated genes & 425 rat T2D-related genes**. Click here for file

Additional file 7 **DCLs identified by DCe**. Click here for file

Additional file 8 **network modules organized by solely correlation reversal**. Click here for file
