# Supplementary material for: Link-based quantitative methods to identify differentially coexpressed genes and gene Pairs
Source: BMC Bioinformatics. 2011 Aug 2;12:315. doi: 10.1186/1471-2105-12-315 (PMC3199761; doi:10.1186/1471-2105-12-315)
Supplement: Additional file 1 — more parameter testing for gene-level evaluation. [file 1471-2105-12-315-S1.DOC]

# Testing more parameter settings of various DCEA methods

# *(supplement to Yu et al.*

# *“differential coexpression analysis”)*

# Testing the threshold for constructing coexpression network and the perturbation rate for simulating differential regulation

As all DCEA methods except WGCNA involve a coexpression network construction step, which is basically determined by a correlation value cut-off, we repeated the performance comparison on various q value cutoffs (0.25, 0.2 and 0.1); additionally, as the perturbation rate was set arbitrarily, we also tried 20% and 30% beside the above 10%. Results were shown in Table 1.

It proved that DCp and DCe consistently outperformed the current methods in all scenarios with DCp doing better than DCe in most situations. We also found that for perturbation rates 10% and 20%, algorithm performances generally increased with more stringent q value cut-offs. While performances of DCp and DCe remained relatively stable for perturbation rates 10% and 20%, they dropped a little at perturbation rate 30%.

**Table 1. AUCs of ROCs of various methods (DCp/DCe/WGCNA/ASC/LRC in turn) tested on two datasets from series A and C respectively. Digits in bold correspond to the two novel methods, DCp and DCe, respectively.**

|  | | Perturbation rate | | | | | |
| --- | --- | --- | --- | --- | --- | --- | --- |
|  |  | 10% | | 20% | | 30% | |
|  |  | All eliminated | Half eliminated,  Half switched | All eliminated | Half eliminated,  Half switched | All eliminated | Half eliminated,  Half switched |
| q_th_ | 0.1 | **0.61**/**0.56**/0.53/0.53/0.55 | **0.69**/**0.62**/0.55/0.48/0.55 | **0.65**/**0.62**/0.54/0.53/0.59 | **0.68**/**0.53**/0.50/0.57/0.54 | **0.59**/**0.6**/0.55/0.48/0.59 | **0.59**/**0.50**/0.51/0.57/0.52 |
|  | 0.2 | **0.60**/**0.55**/0.530.53/0.55 | **0.67**/**0.62**/0.55/0.47/0.54 | **0.63**/**0.60**/0.54/0.54/0.59 | **0.65**/**0.54**/0.50/0.57/0.50 | **0.61**/**0.58**/0.55/0.48/0.56 | **0.57**/**0.51**/0.51/0.55/0.52 |
|  | 0.25 | **0.60**/**0.54**/0.53/0.54/0.53 | **0.66/0.61**/0.55/0.50/0.53 | **0.61**/**0.58/**0.54/0.55/0.58 | **0.64**/**0.55**/0.50/0.57/0.50 | **0.61**/**0.57**/0.55/0.49/0.56 | **0.56**/**0.51**/0.51/0.54/0.52 |

# Testing various setting of the parameter ‘power’ of the signed/unsigned WGCNA

Of the three comparative methods we reviewed, WGCNA has been used most widely, and it does outperform the other two existing methods ASC and LRC. In fact, WGCNA has evolved to two different versions, namely the signed WGCNA and the unsigned WGCNA, and both versions are dependent on a key parameter for tuning the soft thresholding strength, the power beta. For a fair, unbiased and comprehensive comparison of our methods against WGCNA, we performed a systematic analysis of the signed WGCNA and the unsigned WGCNA over a spectrum of the power beta: 1, 2,4,6,8,10,12,18,24, and summarized the results in Fig. 1, Fig. 2, and Table 2. The DCp and DCe performances (q_th_=0.25 and perturbation rate = 10%) were also shown for a comparison. Note that in each scenario five replicated dataset pairs were used to repeatedly test each method.

It turned out that in general the signed WGCNA was more powerful than the unsigned WGCNA in terms of differential coexpression analysis, but yet both were incomparable to DCp and DCe regardless of the choices of the parameter beta (Fig. 1 and Fig. 2). In addition, we found that the WGCNA methods were more competent for the regulation-removal scenario, in constrast to DCp and DCe who excelled in the regulation-toggle scenario (Fig. 2). Finally, the power of discriminating differentially regulated genes of WGCNA methods deteriorates with the beta value, especially when the beta exceeded eight (Fig. 2 and Table 2).


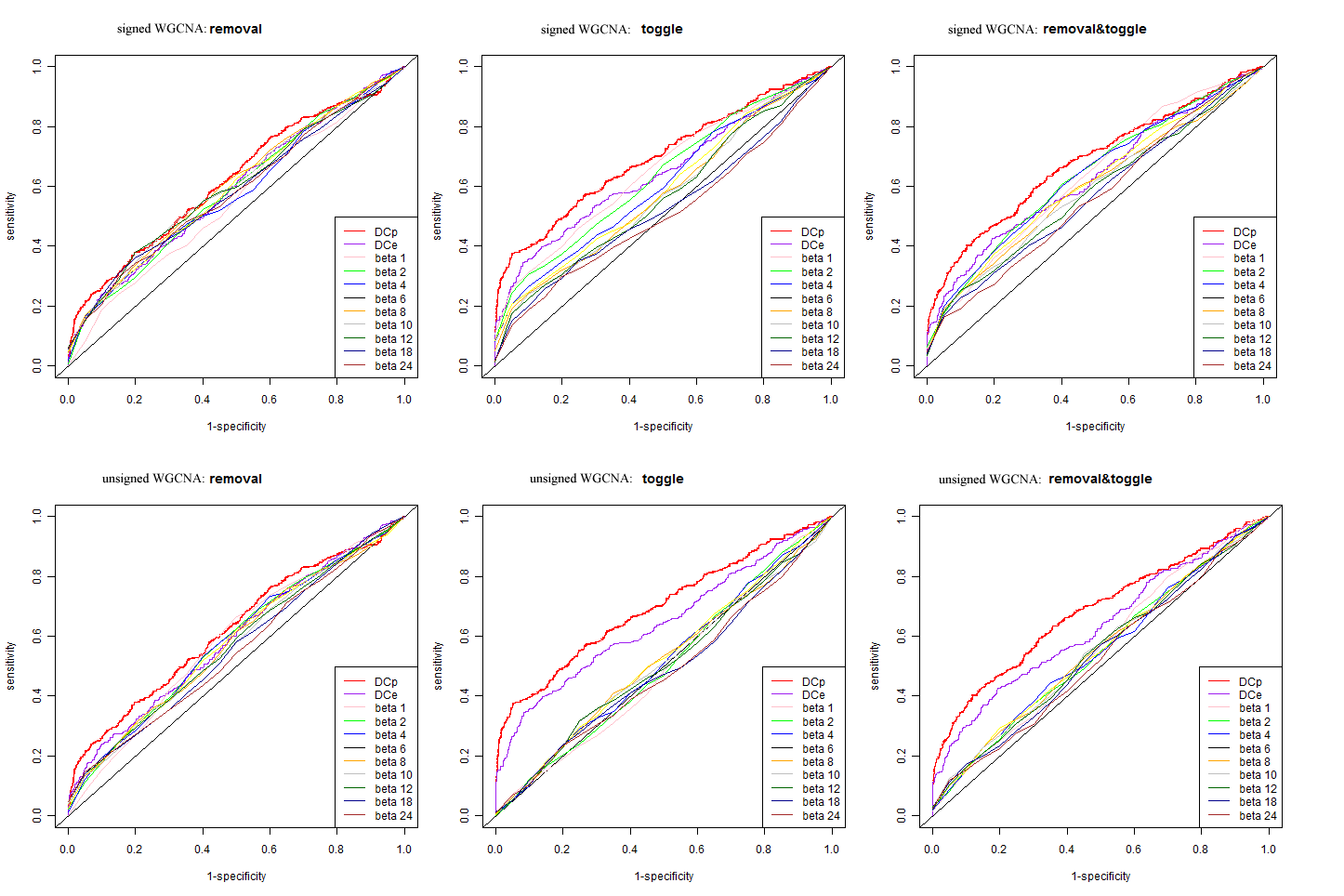


Figure 1. Receiver-Operating Characteristic curves of various parameter settings of signed WGCNA (upper panel) and unsigned WGCNA (below panel). The two novel methods DCp and DCe are also shown for a comparison.


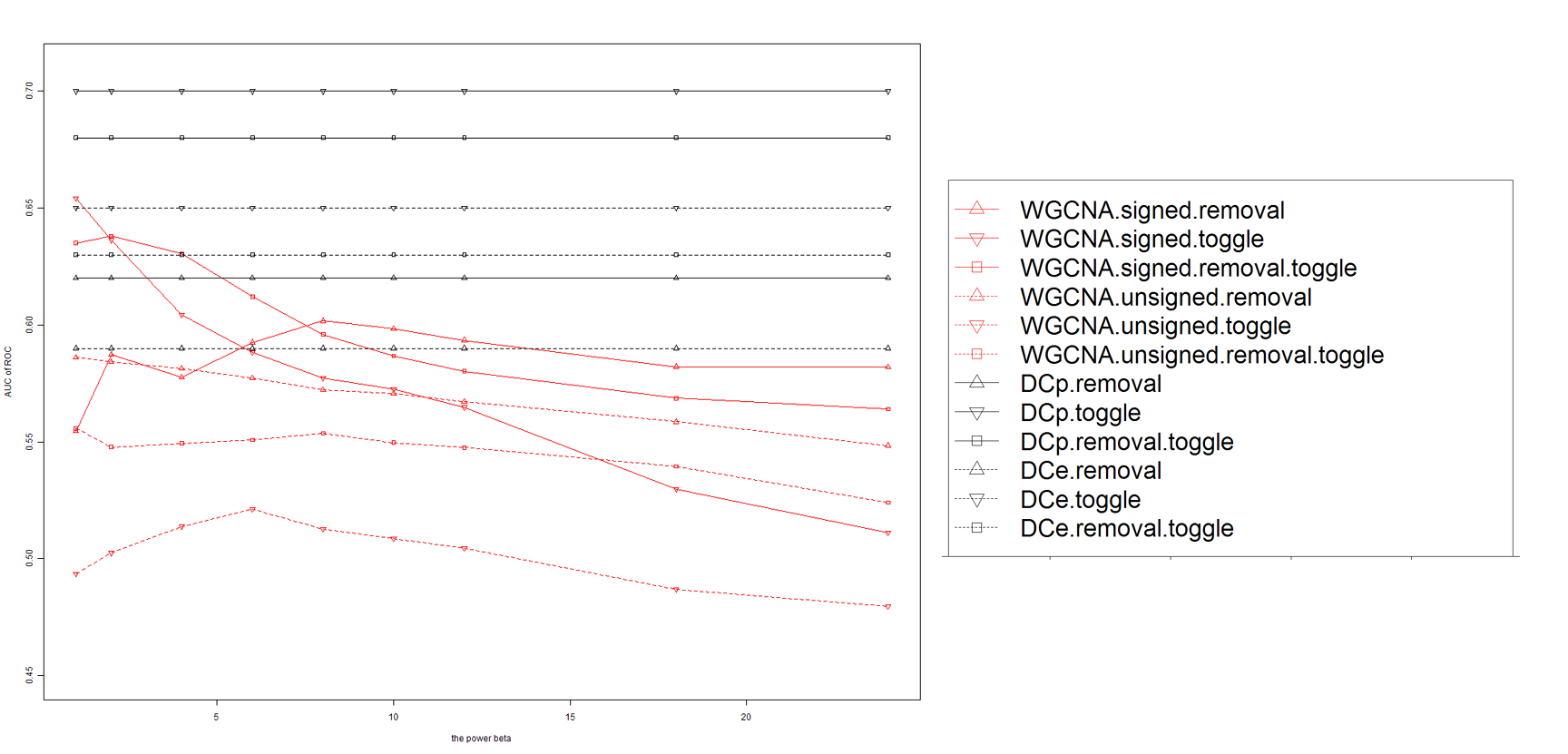


Figure 2. Areas under curve (AUCs) of the ROCs of signed WGCNA and unsigned WGCNA in relation to values of the parameter beta (refer to Fig.1). DCp and DCe are also shown for a comparison. Note that each method is associated with three lines designating the three network perturbation types respectively.

Table 2. Mean and the standard deviation of AUCs of signed/unsigned WGCNA over five replicated tests. Each method is tested against nine values of the power beta (rows), and three different perturbation types (columns).

| **The power beta** | **Signed removal** | **Signed toggle** | **Signed removal&toggle** | **Unsigned removal** | **Unsigned toggle** | **Unsigned removal&toggle** |
| --- | --- | --- | --- | --- | --- | --- |
| 1 | 0.554+-0.016 | 0.654+-0.008 | 0.635+-0.009 | 0.586+-0.015 | 0.493+-0.020 | 0.555+-0.016 |
| 2 | 0.587+-0.033 | 0.636+-0.002 | 0.638+-0.013 | 0.584+-0.012 | 0.502+-0.018 | 0.547+-0.010 |
| 4 | 0.577+-0.027 | 0.604+-0.021 | 0.630+-0.014 | 0.581+-0.016 | 0.513+-0.012 | 0.549+-0.015 |
| 6 | 0.592+-0.035 | 0.588+-0.024 | 0.612+-0.014 | 0.577+-0.021 | 0.521+-0.005 | 0.550+-0.014 |
| 8 | 0.601+-0.040 | 0.577+-0.023 | 0.595+-0.015 | 0.572+-0.023 | 0.512+-0.003 | 0.553+-0.012 |
| 10 | 0.598+-0.039 | 0.572+-0.018 | 0.586+-0.017 | 0.570+-0.025 | 0.508+-0.000 | 0.549+-0.012 |
| 12 | 0.593+-0.036 | 0.564+-0.016 | 0.580+-0.017 | 0.567+-0.024 | 0.504+-0.000 | 0.547+-0.011 |
| 18 | 0.582+-0.028 | 0.529+-0.018 | 0.568+-0.012 | 0.558+-0.024 | 0.486+-0.003 | 0.539+-0.008 |
| 24 | 0.581+-0.028 | 0.511+-0.016 | 0.564+-0.015 | 0.548+-0.018 | 0.479+-0.005 | 0.524+-0.000 |

The signed WGCNA method is more powerful than the unsigned WGCNA method in terms of performing differential coexpression analysis. This is consistent with their different designs – crushing [-1,1] values to a narrower region [0,1] reserves more information for negative correlations than simply flipping them to the opposite side. However, as we illustrated above, the signed WGCNA does not lay as equal importance on negative correlation values as on positive correlation values. Perhaps due to this point, the signed WGCNA turned out to be incomparable to DCp and DCe (Fig.1 and Fig.2).

When inspecting the signed or unsigned WGCNA over the spectrum of beta values, we found that in general the differential coexpression analysis performance decreases with the beta value, especially when the beta exceeded eight (Fig. 2 and Table 2). In addition, we found that the WGCNA methods were more competent for the regulation-removal scenario, in constrast to DCp and DCe who excelled in the regulation-toggle scenario (Fig. 2).

# Sample-size testing

We learned from a reference (Choi and Kendziorski, 2009) that sample size less than five is unreliable for correlation analyses. We tested the algorithm performances in simulation datasets with sample sizes varying at five, six, seven, eight, nine, ten, fifteen and twenty. It was shown that the performances of all algorithms are generally insensitive to the sample size variation and that DCp and DCe were better than the other methods at the most time (Figure 3, A, B, and C). So users do not need to worry about the sample size as long as it exceeds a minimum of five.

Figure 3A. Area Under Curves of five methods at different sample sizes. Simulated datasets were originated from a dataset pair in Group A (pure regulation-elimination).

Figure 3B. Area Under Curves of five methods at different sample sizes. Simulated datasets were originated from a dataset pair in Group B (pure regulation-switch).

Figure 3C. Area Under Curves of five methods at different sample sizes. Simulated datasets were originated from a dataset pair in Group C (half regulation-elimination and half regulation-switch).
