# Supplementary material for: Link-based quantitative methods to identify differentially coexpressed genes and gene Pairs
Source: BMC Bioinformatics. 2011 Aug 2;12:315. doi: 10.1186/1471-2105-12-315 (PMC3199761; doi:10.1186/1471-2105-12-315)
Supplement: Additional file 2 — significance in Link-level evaluation. [file 1471-2105-12-315-S2.DOC]

# DCe retrieves significantly many DRLs compared with random guess

# *(supplement to Yu et al.*

# *“differential coexpression analysis”)*


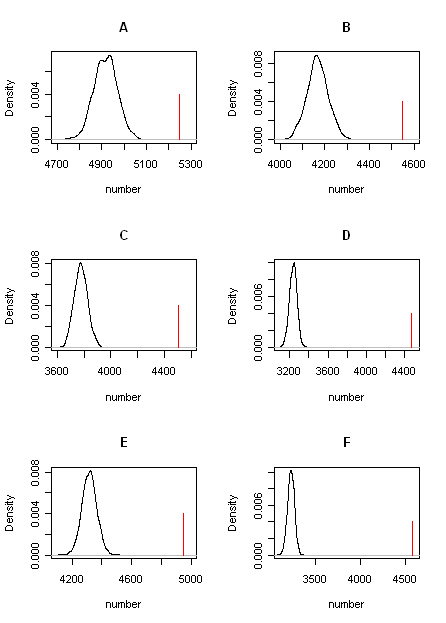


Dataset I: same-signed

Dataset II: same-signed

Dataset III: same-signed

Dataset I: differently-signed

Dataset III: differently-signed

Dataset II: differently-signed

**Supplementary Figure. DCe fetches a significantly larger number of extended DRLs than random manipulations.** Red bars indicate the number of extended DRLs in DCe results, while the density plot shows the distribution of the number of extended DRLs in random sampled links of equal size. The random sampling experiment was repeated 1000 times for each link part (left column: same-signed links; right column: differently-signed links) of each dataset pair separately (1^st^ row: a dataset pair (I) from group A; 2^nd^ row: a dataset group (II) from group B; 3^rd^ row: a dataset pair from group C). For definition of ‘extended DRLs’, see related text in the manuscript.
