# Supplementary material for: Link-based quantitative methods to identify differentially coexpressed genes and gene Pairs
Source: BMC Bioinformatics. 2011 Aug 2;12:315. doi: 10.1186/1471-2105-12-315 (PMC3199761; doi:10.1186/1471-2105-12-315)
Supplement: Additional file 3 — reducing DCL scales. [file 1471-2105-12-315-S3.DOC]

# Scaling down DCLs by tuning algorithm parameters

# *(supplement to Yu et al.*

# *“differential coexpression analysis”)*

Showing the most outstanding performance in retrieving predefined differentially regulated links (DRLs), DCe is recommended as the priority method for this task. In reality, people may wish to scale down the identified DCLs for a focused follow-up examination. For this purpose, one can raise coexpression value cut-offs (ρ) or lower outlier fractions (δ). We found that raising ρ refined correlation-reversed DCLs efficiently (Fig. S1 in this document) while lowering δ not only reduced the scales of DCLs of the other two types (same-signed and differently-signed) but promoted the accuracy of identified DCGs (Table S1 in this document).


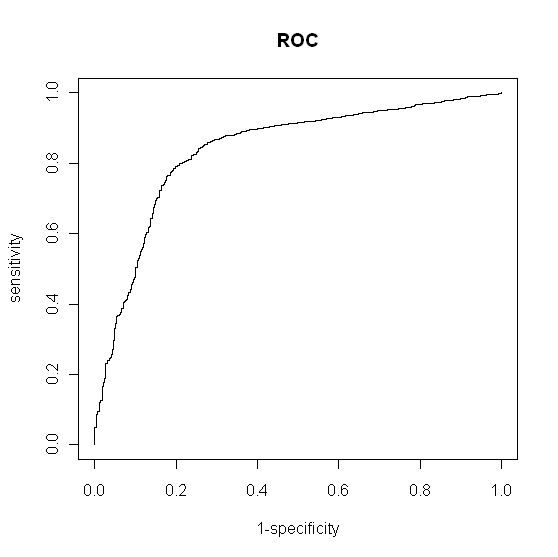


**Figure S1. The ‘maximum absolute correlation’ cut-off (**ρ**) is an efficient parameter for refining the set of correlation-switched DCLs.** From bottom-left to upper-right, ρ was lowered gradually to determine enlarged sets of correlation-switched DCLs. Taking the ‘extended DRLs’ (Definition found in the manuscript) as the golden standard DCLs, we observed the specificity of DCL discremination dropped with the increase of sensitivity. A convex Receiver-Operating-Characteristic curve indicates the discriminability of the tuning parameter ρ**.**

# Table S1.Results were scaled down while precision of DCG identification was promoted with the lowered ‘outlier fraction’ (δ) of the LFC model. Data were reported for a simulated dataset in Series C (see explanation in Methods 2.3)

| $\delta$ | **# DCGs** | **# extended DRLs** | **DCG Accuracy** | **DCLs*** | **DCLs* per DCGs** |
| --- | --- | --- | --- | --- | --- |
| **0.1** | **89** | **58** | **0.65** | **9334** | **105** |
| **0.05** | **73** | **54** | **0.74** | **4342** | **59** |
| **0.01** | **48** | **43** | **0.90** | **831** | **17** |

the correlation-switched type was excluded from these DCLs; i.e., DCLs here reported comprised the same-signed and the differently-signed DCLs.
