# Supplementary material for: Link-based quantitative methods to identify differentially coexpressed genes and gene Pairs
Source: BMC Bioinformatics. 2011 Aug 2;12:315. doi: 10.1186/1471-2105-12-315 (PMC3199761; doi:10.1186/1471-2105-12-315)
Supplement: Additional file 4 — Preprocessing GSE3068. [file 1471-2105-12-315-S4.DOC]

# Preprocessing GSE3068 dataset

# *(supplement to Yu et al.*

# *“differential co-expression analysis”)*

A public rat diabetes-or-normal gene expression dataset was obtained from Gene Expression Omnibus (GEO) database (http://www.ncbi.nlm.nih.gov/geo/) with accession number GSE3068. This dataset is generated with the Affymetrix RG-U34A chip, originally included 8799 probesets. For ease of the following analysis, we deleted probesets mapping to no gene ID or multiple gene IDs, which left 6,955 probesets for following data analysis. In the case of situation of multiple probesets mapping to one identical gene, we followed the following rules to ensure there was only one probeset left to interrogate one gene:

1. Prioritize probesets according to the suffixes in the order shown in Table 1, and remove all probesets except the highest-ranked one. The priority is determined according to the ‘Affymetrix GeneChip Expression Analysis Data Analysis Fundamentals’ (<http://www.affymetrix.com/support/downloads/manuals/data_analysis_fundamentals_manual.pdf>).
2. If there are multiple top-ranked probesets remained for a gene, keep the probeset which is most often associated with the highest expression level.
3. If there are still more than one probesets left after the step 2, average the intensities of the remaining probesets. [Only one gene came to rule 3, which was EntrezGene 54243. Its expression intensity was an average of two probesets: D42116_s_at and L36532_s_at]

Table 1. Priority order of probeset suffixes in RG-U34A chip

| probeset | priority |
| --- | --- |
| _at | 1 |
| _f_at | 2 |
| _s_at | 3 |
| _g_at | 4 |
| _i_at | 5 |
| _r_at. | 6 |

As shown in Table 2, 67.8% of all genes (3232, precisely) were each interrogated by only one probeset, therefore retained their original information in our preprocessing; another 20.3% (963) genes were each interrogated by only one ‘_at’ probeset, whose unique representatives should unambiguously be their only ‘_at’ probesets. In summary, 11.9% of all genes were subjected to our suffix ranking and expression-based voting procedures. Finally, a one-to-one match from 4765 probesets to 4765 genes was achieved.

Table 2. Genes on RG-U34A classified according to their probeset suffixes.

|  | | Numbers | Fractions |
| --- | --- | --- | --- |
| Singleton genes | Interrogated by _at probeset | 2842 | 59.6% |
|  | Interrogated by other extension | 390 | 8.2% |
|  | ***Subtotals*** | ***3232*** | ***67.8%*** |
| Redundant genes | Interrogated by only one _at probeset | 963 | 20.3% |
|  | Interrogated by two or more _at probesets | 424 | 8.8% |
|  | Not interrogated by _at probeset | 146 | 3.1% |
|  | ***Subtotals*** | ***1533*** | ***32.2%*** |
| Altogether |  | 4765 | 100% |
