# Supplementary material for: Link-based quantitative methods to identify differentially coexpressed genes and gene Pairs
Source: BMC Bioinformatics. 2011 Aug 2;12:315. doi: 10.1186/1471-2105-12-315 (PMC3199761; doi:10.1186/1471-2105-12-315)

**Correlation reversion network modules**

***(supplement to Yu et al. “differential coexpression analysis”)***

The following figures embody gene network modules connected with 110 normal-to-T2D correlation-reversed gene links. Green and brickred circles denote DCGs and non DCGs, respectively. A blue link connects a pair of genes whose positive correlation in normal state was switched to a negative one in the T2D disease state, while a red link indicates a correlation reversion in the opposite direction, i.e., from negative (normal) to positive (disease). These results were obtained from analysis of the GEO GSE3068 dataset. For more information, see other additional files of this manuscript.


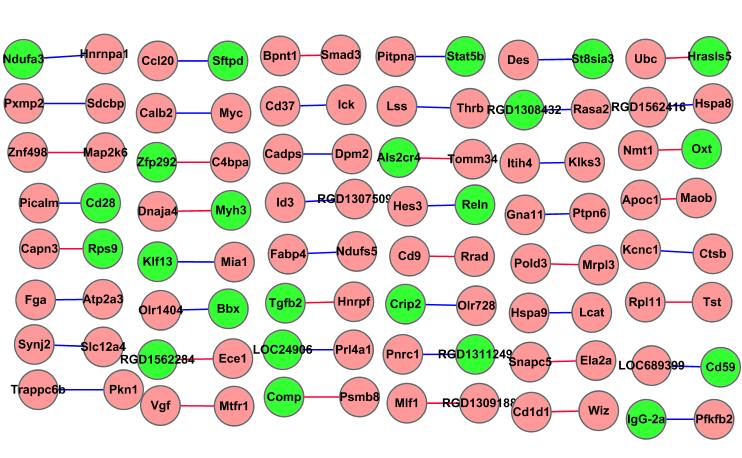


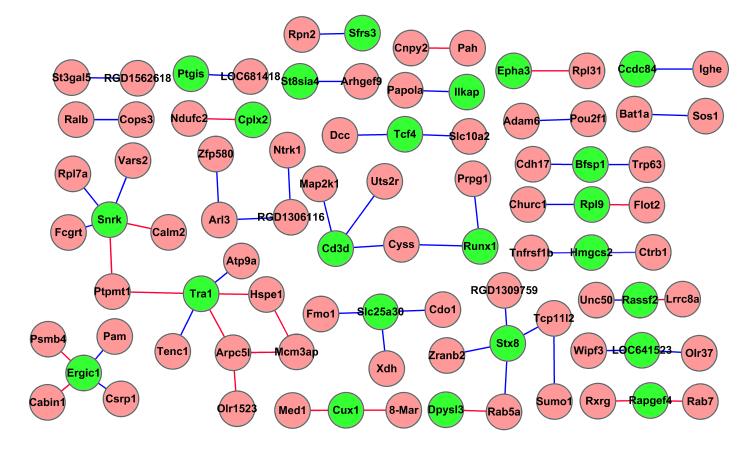

Supplement: Additional file 8 — network modules organized by solely correlation reversal. [file 1471-2105-12-315-S8.DOC]
